# The Epidemiology of Malaria in Kutubu, Southern Highlands Province, Papua New Guinea, before and during a Private Sector Initiative for Malaria Control

**DOI:** 10.3390/tropicalmed2010002

**Published:** 2017-02-10

**Authors:** Marshall Feterl, Patricia Graves, Liesel Seehofer, Jeffery Warner, Peter Wood, Kevin Miles, Ross Hutton

**Affiliations:** 1 Australian Institute of Tropical Health and Medicine, Division of Tropical Health and Medicine, James Cook University, Townsville 4811, Australia; marshall.feterl@jcu.edu.au (M.F.); patricia.graves@jcu.edu.au (P.G.); jeffrey.warner@jcu.edu.au (J.W.); 2 College of Public Health, Medical and Veterinary Sciences, Division of Tropical Health and Medicine, James Cook University, Cairns 6811, Australia; peter.wood@jcu.edu.au; 3 Oil Search Foundation, P.O. Box 842, Port Moresby, Papua New Guinea; lieselseehofer@yahoo.com.au (L.S.); ross_hutton@bigpond.com (R.H.)

**Keywords:** malaria, Papua New Guinea, Kutubu, Southern Highlands, social marketing, EDAT, public-private partnerships

## Abstract

Papua New Guinea (PNG) has a significant malaria burden, is resource constrained, and has isolated populations with limited access to health services. Home-based management is a key element of the national program that supports strategies of early detection, diagnosis and treatment. We describe the epidemiology of malaria near Lake Kutubu in the Southern Highlands Province through reported data on suspected and confirmed malaria in patients accessing public health facilities or using a novel, incentivised, social marketing approach for malaria treatment at the village level. Monthly case data reported by nine health facilities and 14 village-based providers, known as Marasin Stoa Kipas (MSK), were extracted from outpatient registers and MSK malaria case forms. Descriptive statistics of diagnostic use, monthly incidence, test positivity rate and species distribution were estimated. Summary statistics of service delivery demonstrate patient access and diagnostic coverage in program areas. From May 2005 to September 2013, 15,726 individuals were tested with either rapid diagnostic test and/or microscopy at health facilities, and 42% had a positive result for malaria (*n* = 6604); of these 67.1% (*n* = 4431) were positive for *P. falciparum* (alone or mixed) and 32.9% were positive for non-*P. falciparum* species (alone or mixed). From October 2007 to September 2013, 9687 individuals were tested with either RDT and/or microscopy at MSK sites and 44.2% (*n* = 4283) tested positive for malaria; of these, 65.3% (*n* = 2796) were positive for *P. falciparum*, while 34.7% (*n* = 1487) were positive for non-*P. falciparum* species. Up to April 2010 there was an intermittent and upward trend in the reported incidence of all species of confirmed malaria, reaching 50 per 1000 population per month for both sites combined, followed by a steady decline to four per 1000 population per month in 2013, with *P. vivax* the most common infection. This study is the most recent longitudinal overview of malaria in the Southern Highlands since 2003. It outlines patient access to a community-based model of care. The analysis shows changes in health facility versus MSK use, a strongly decreasing trend in incidence of confirmed malaria from 2010 to 2013, and a shift from predominantly *P. falciparum* to *P. vivax* infection.

## 1. Introduction

Malaria remains one of the largest public health burdens in Papua New Guinea (PNG) [1] and is one of the leading causes of morbidity and mortality, with a reported incidence in 2015 of 102 per 1000 population per year [2]. According to the PNG National Department of Health (NDoH), 90% of the population is at risk of malaria, with *Plasmodium falciparum* (56%) and *Plasmodium vivax* (41%) the most common species [3]. However, there is large geographic heterogeneity in species and risk of infection. 

Malaria admissions and mortality have declined appreciably over the past five years due to long-lasting insecticide-treated nets (LLIN), rapid diagnostic tests (RDT) and first-line antimalarial distribution and use [4,5,6,7]. Provision of preventive interventions (LLIN) is crucial [4,5], but sustaining effective control measures is also dependent on strong health systems capable of delivering reliable diagnostics and treatment, particularly in remote locations. 

Prior to large-scale surveys conducted by the PNG Institute of Medical Research (PNGIMR) from 2000 to 2005 [8,9,10], historical epidemiological studies of malaria in the PNG highlands were fuelled by industry as early as the 1940s, when control efforts were centred around economically important areas [8]. This phenomenon holds true in the resource sector today, where exploration and development can import labour from endemic regions both domestically and internationally [11,12]. The National Malaria Programs of the late 1970s up to the 1990s never extended into the most remote development regions in the Western and Southern Highlands [13,14]. Hii and colleagues conducted a series of prevalence surveys in the Tagari Valley of the Southern Highlands in 1990 and 1991 as part of a health assessment and risk analysis prior to the development of British Petroleum (BP) and later Oil Search Limited’s (OSL) Hides Gas Field project [11]. Surveys indicated that prevalence rates decreased with altitude (48% to 5%). *P. falciparum* was the dominant species at lower elevations, while the proportion of infection attributed to *P. vivax* increased at altitudes above 1700 m. As a result of the assessment BP, and later OSL, now PNG’s largest oil and gas producer, provided occupational and community based malaria control programs to protect the workforce and provide benefits for local communities. At the time of this study, community health programs were delivered by the Oil Search Public Health Unit. The community health operations of Oil Search have since developed into the Oil Search Foundation, a unique arm of the company’s sustainable development strategy. 

Papua New Guinea has a challenging topography and limited infrastructure, with 85% of the population living in rural environments. Isolated populations and complications in delivering managerial and logistical support to health services are two of the major barriers to providing effective diagnosis and treatment for most communicable diseases. Prior to the National Malaria Treatment protocol being implemented in 2009, less than 20% of patients had received a diagnostic test when presenting with febrile illness or malaria-like symptoms at public sector providers [15]. In 2014, household survey results indicated that 43.5% of children under five and 42.6% of those over five years attended a health facility for recent fever episodes in PNG, while 17% were given an appropriate test [16]. Of those testing positive, 78% received an appropriate artemisinin combination-based therapy (ACT) as per the national treatment guidelines. Significant deficiency in appropriate treatment delivery is thus experienced in PNG, where only 40% of surveyed providers dispensed treatment consistent with national guidelines in 2012 [16]. In a recent study assessing health worker compliance to ‘test and treat’ protocols outlined in the PNG NDoH National Guidelines, 77.6% of patients with fever or suspected malaria were tested by a rapid diagnostic test (RDT) or microscopy, 65.6% of confirmed malaria cases were prescribed the correct ACT and 15.3% of febrile patients who tested negative were incorrectly prescribed antimalarials [17]. While the availability of ACT drugs in peripheral centres has risen, there are limited data on appropriate usage. A supervised system that provides improved early access to detection, diagnosis and treatment in accordance with national guidelines at the peripheral level is needed to supplement the formal health system. In this study, we review a community-based malaria control program in Kutubu, Southern Highlands Province, initiated as a unique partnership between a private company and national and local government.

**Figure 1:**
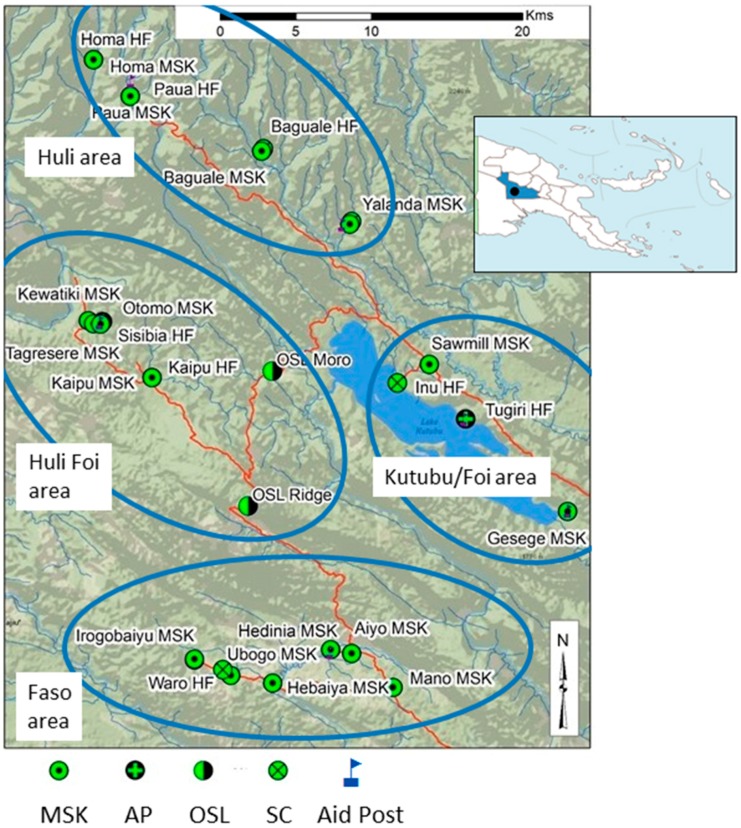
Map of villages and ethnic clusters around Lake Kutubu, SHP. Inlaid map of PNG at top right; SHP indicated in blue; (●) location of Moro on inlaid map; base of OSL public health lab and malaria program field office.

In the Southern Highlands the Nipa-Kutubu District (area 6794 km^2^) is the largest of five districts with a population of 153,986 according to the PNG National 2011 Census [18]. At the time of the study, the Oil Search malaria program serviced a catchment population of approximately 10,850 according to PNG district profiles [19]. Elevation in Nipa-Kutubu ranges from 391 m to 1374 m. There is little seasonal variation in rainfall with averages of >200 mm per month during the wet season to 100–200 mm in the dry season (July to October) [20]. The Kutubu region was classified as a highly endemic area for malaria by the PNG IMR (17%–33% prevalence) in 2003, with all four *Plasmodium* spp. present [10]. For the purposes of this paper, “Kutubu” defines the study area outlined in Figure 1.

The base of OSL community malaria operations at the time of the study was located in Moro, which is in a low-lying intermountain region near the shores of Lake Kutubu (Figure 1). Initial assessments of local health facility performance determined that health systems were failing, and malaria was the most significant cause of morbidity and mortality in febrile patients. As a response, OSL, in partnership with government and church-based partners, implemented a novel village-based malaria control program whereby an incentivised social business model combined with social marketing techniques was developed to train local community members to provide early diagnosis and treatment (EDAT) for malaria and improve treatment-seeking and completion of appropriate full treatment courses. The strategy was called the “Marasin Stoa Kipa” Project (MSK) in Tok Pisin, the local lingua franca, which translates to “Medicine Store Keeper”. The program implemented stringent quality control measures for diagnosis by providing a RDT for all fever cases at the MSK site, where they were cross-checked against blood smear results by OSL microscopists. Any discrepancies between RDT and blood smear results were flagged and patients were retested. Patients with a positive result were administered age- and weight-adjusted dosages of antimalarials by the MSK, who adhered to National Malaria Program recommended guidelines.

Here, we outline the results of monthly malaria case data collected from May 2005 to September 2013 from nine government- and church-managed health facilities (HF) and 16 village-based MSK sites to describe the epidemiology of confirmed malaria in Kutubu. Surveillance data collected from these services (both RDT and blood smears) were used to describe patient access of both HF and MSK.

## 2. Methods

### 2.1. Intervention: Marasin Stoa Kipa (MSK): 2007–2013

The initial strategy to reduce mortality and morbidity focused on strengthening existing health systems, namely aid posts and health centres. After two years the church-run centres functioned reasonably well due to improved supervision and staff management, but government centres remained challenged. 

The lesson learned from the years 2005–2007 was that developing and maintaining a salaried health worker in remote areas was problematic. Oftentimes workers were not from the region, so were far from their families and cultural support and not motivated to work. Workers continued to be paid despite long absences. The aforementioned factors, among others, contributed to a high degree of absenteeism and disruption of service.

From 2007–2010 OSL developed and tested the MSK strategy to serve as an adjunct to malaria control in its Kutubu program area. Six key strategies underpinned the program:

(1) implement the MSK program in malaria endemic communities with no functional health facilities or access to care;
(2) implement the MSK program in areas where health facility function was challenged;
(3) motivate the MSK with financial incentives according to quantity and quality of work;
(4) embed local management of the MSK program with a community-based organization, such as the church;
(5) develop the MSK as an integral component of local health services (public private partnerships);
(6) develop a monitoring and evaluation (M&E) system to evaluate progress and adjust elements of the program according to specific community needs where applicable.

Village-based MSK providers were recruited locally against key selection criteria to provide early diagnosis and treatment to community members. To emphasize that they were not unpaid volunteers but private entrepreneurs, they were called MSKs. Among other criteria, MSKs had to be permanent and respected local residents, to have achieved a level of literacy, and to preferably be married females, for cultural reasons. This reduced the likelihood of a high turnover, due to the customary practice of moving to the husband’s village upon marriage. In addition, young unmarried women faced greater community resistance to seeing male patients, particularly if unchaperoned.

The MSKs were trained, supplied and supervised by the OSL Public Health Service. They were given malaria kits for storage of all supplies and equipment for recording, diagnosing and treating malaria. 

Standard treatment provided by health centres at the commencement of the program included a combination of chloroquine/sulphadoxine-pyrimethamine for adults and amodiaquine/sulphadoxine-pyrimethamine for children in eight weight categories. The three-day course of tablets was usually dispensed in small plastic bags or wrapped in paper. Health workers often only dispensed medication one day at a time, requiring patients to return to the health centre daily to ensure patients complied with the three-day regimen. 

The OSL Public Health Service pre-packages treatment into blister packs according to dose for weight category using a locally produced blister packaging machine. In an effort to simplify the treatment regimen and improve compliance, the weight categories for treatment doses were reduced from eight used at health facilities to five following consultation with the PNG Paediatric Society and pharmacologists in accordance with PNG National Guidelines. Each blister pack was placed in an attractive outer box that was colour-coded to clearly show the corresponding weight/age group and pictorial instructions for use. Indexes on weighing scales were also colour-coded to facilitate the dispensing of the correct package and dosage. There were iterations of the treatment regimen, and antimalarial, dosage and colour scheme changed over time to remain in compliance with National Malaria treatment guidelines. In 2011–2012, PNG malaria treatment guidelines shifted to the use of ACTs and the treatment was subsequently implemented in the MSK program. 

In comparison to the standard care treatment available in Kutubu, the MSK program ensured an optimal service using strict quality control measures, direct supervision and monetary incentives. To enable immediate diagnosis, RDTs were provided (Immunocomb, Alere, Australia). MSKs were also taught to collect and stain thick and thin blood smears to enable quality assurance microscopy in the Oil Search laboratory for surveillance and monitoring purposes. Initially, an incentive of 40 Kina (12.62 USD) as a fixed monthly allowance was given to the MSK for collection of quality blood smears and recording of data on surveillance forms. Deductions in the monthly allowance were made in the event of reporting errors and or poor blood films to further incentivize quality service provision. Later, a service fee was charged to each patient in exchange for the monthly allowance and a flat rate of 1 Kina per quality blood smear to facilitate greater income stream. The treatment program was subsidized by OSL to remove cost prohibitive barriers and to assess the proof of concept of improving EDAT. Social marketing of the concept to local communities was carried out by preparing individualized promotional posters of each MSK for display on poster boards and walls in the community. OSL Public Health Officers explained and discussed the program with the community in several meetings. Church pastors were trained to promote and support the MSK. 

The OSL Public Health Officers initially visited weekly, then bi-weekly and later monthly, to ensure supply stocks were adequate to meet demand, monitor standards and quality, and provide additional training if required. Replacement stock was given according to the number of packages consumed. Cross-checks of case detection forms matching the blood slides that were collected and the number of consumed drug packages according to weight category helped improve quality assurance. Malaria incidence was monitored with RDT and blood slides collected by the MSK from each patient. Because MSKs did not have alternative treatment, they had no choice but to record and test all patients they provided with malaria treatment. This procedure resulted in recording and blood sampling 100% of patients treated for suspected malaria. The blood slides were examined in the OSL-PHS lab in Moro. In addition, biannual cross-sectional prevalence surveys were carried out in most villages from 2007 to 2010, with interruptions in 2010–2012. Further studies analysing the prevalence data will be reported separately.

Locations of health centres and MSK sites were obtained from The National Research Institute of Papua New Guinea [21] and plotted onto shape files obtained from Vector Map Level online database, United States Defense Mapping Agency (Highlands Ranch, CO, USA) [22]. 

### 2.2. Data on Reported Cases

Patient details were recorded on case detection forms provided to the MSK by the OSL malaria program field officers; (name, sex, age, weight, address, where the patient slept two weeks prior to symptoms, RDT result and treatment administered were recorded). Capillary blood samples were collected from suspected malaria patients by finger stick and diagnosis made by RDT (Immunocomb). One thick and one thin blood film were prepared for each patient. Slides were fixed in methanol and thin and thick films were stained in 10% Giemsa by the MSK. Slides were placed in ziplocked bags, stored at room temperature and placed in a pouch with the facility name. These pouches were collected by OSL Public Health officers fortnightly. Slides were read at the OSL laboratory by light microscopy by a trained microscopist. A minimum of 200 thick film fields were read before declaring a slide negative. The number of parasites was counted against white blood cells until reaching 200 white blood cells. 

At the time of the study, OSL operated on a 28-day rotation for each microscopist and monthly blind cross-checks of a minimum of 80 slides were carried out by the incoming microscopist during each handover period. Any discordant slides identified were read again by both microscopists and, if no consensus was made, the slide was sent to a third party for confirmation by the program coordinator. Malaria data for each individual case seen at HF (outpatients register, case detection forms) and MSK case detection forms were transcribed into a Microsoft Excel database (Microsoft Corporation, Seattle, WA, USA). 

The data covered the time period from May 2005 to September 2013 with a total of 28,819 individual patient records accessing 25 treatment provision sites (16 MSK, 9 HF). Data were cleaned using range checks, spelling variations and examination of missing values, and collapsed to monthly summaries of number tested, number positive (any species) and incidence by site. The test result variables used were number positive by any test, number missing, negative or positive by malaria species (or mixed) by microscopy, and number positive (*P. falciparum* only, Pf mixed, other *Plasmodium*), negative, invalid or missing by RDT. Age in years was assigned to age groups of <1, 1 to <5, 5 to 10, 10 to 15, 15 to 20, 20 to 30, 30 to 40, 40 to 60, and 60 to 100 years. Gender was classified as male, female or unknown (based on incomplete or missing records). Population data to determine malaria incidence of villages in the OSL study area were provided by census data collected by the OSL department of community affairs. 

Original RDT and microscopy results were recorded from patient registries and case detection forms collected from HF and MSK. Details of original RDT and microscopy reporting can be viewed in the Supplementary Materials. 

## 3. Results

### 3.1. Access and Use of HFs and MSK Providers

The data on number of tests performed were used as a measure of patient access to MSK services, the test positivity rate, and to identify the proportion of infections attributed to *P. falciparum* and non-*P. falciparum* species. A summary by year of the number of open HFs or MSK sites, the reporting time periods and the number of individuals tested from 2005 to 2013 for HF and 2007 to 2013 for MSK by RDT, microscopy, or both are reported separately for HF (Table 1) and MSK (Table 2). A total of 1179 facility months of observation were recorded from May 2005 to September 2013, 559 months at HF and 620 months at MSK. The maximum and minimum number of reporting months per annum ranged from 26 to 90 for all HF and 20 to 129 for all MSK.

**Figure 2:**
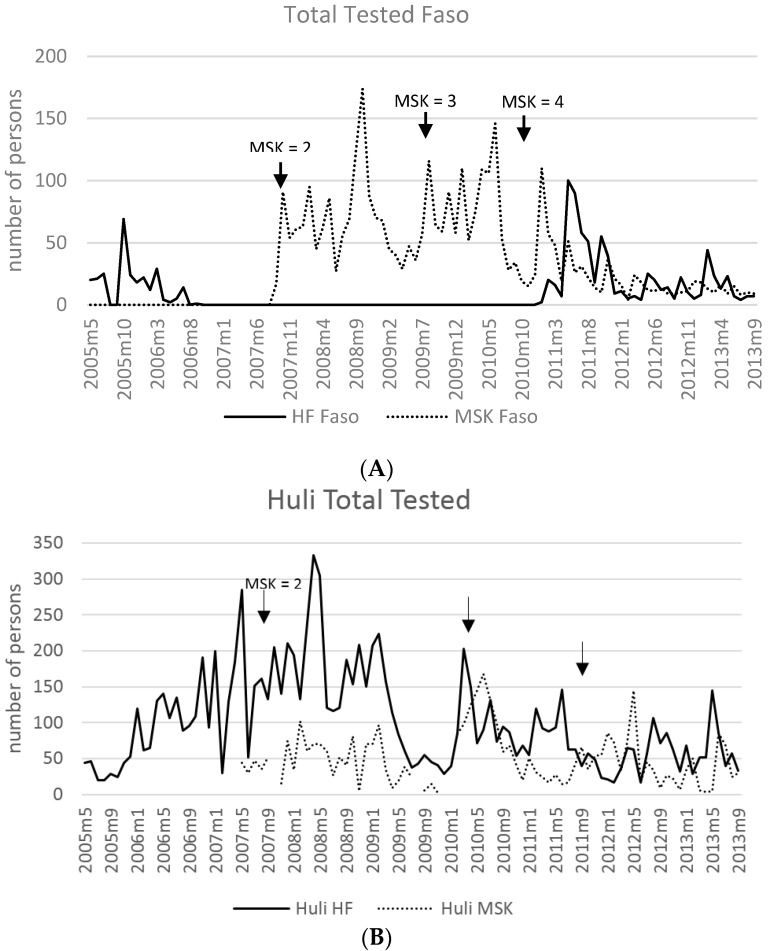

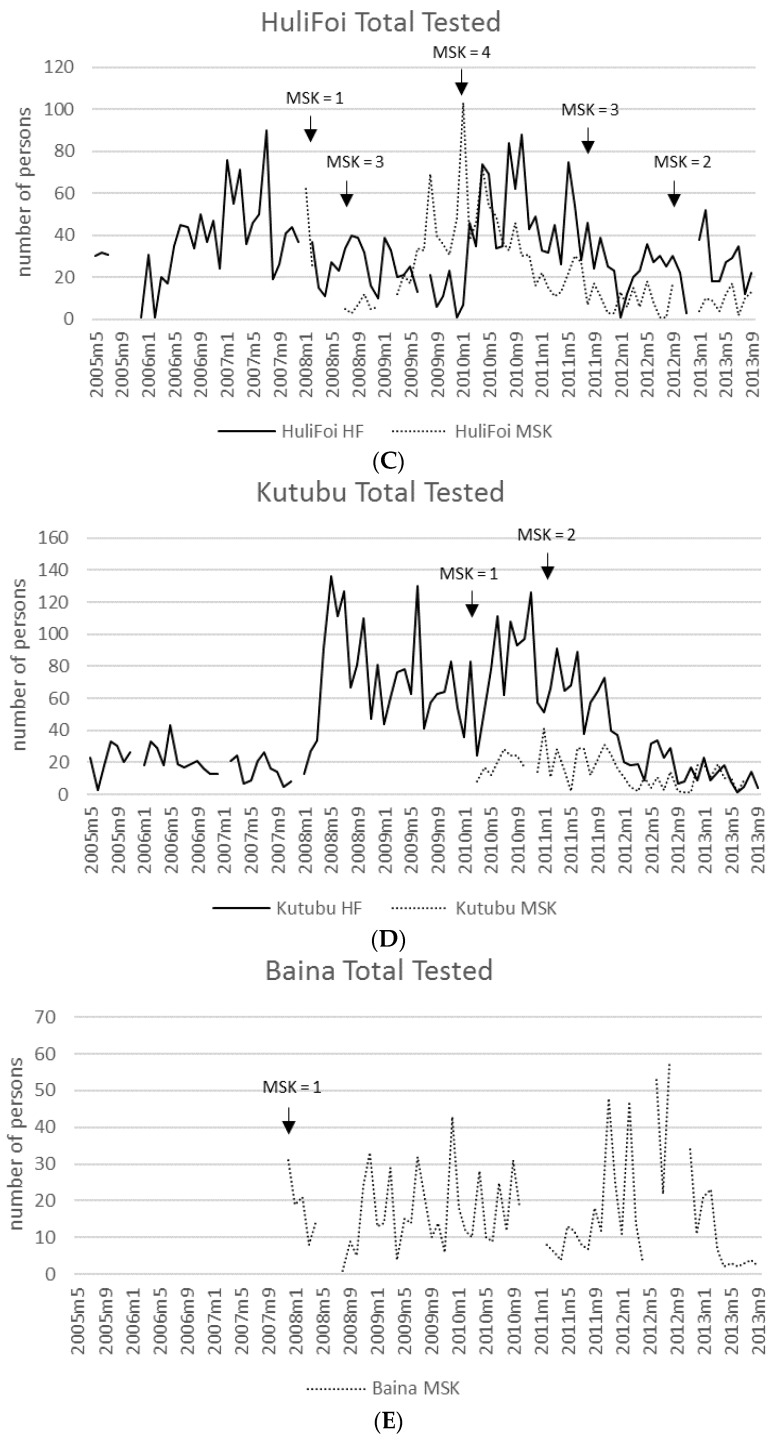
Number of persons tested with RDT and/or microscopy at MSK and HF by ethnic cluster: (**A**) Faso; (**B**) Huli; (**C**) Huli-Foi; (**D**) Kutubu; (**E**) Baina. The rollout of the first MSK site in each cluster and the total number of operating MSK sites are indicated along the timeline.

In Figure 2, an additional stratified measure of patient access and use of the MSK service is provided. A summary of the total number of individuals tested by RDT, microscopy, or both per ethnic area (Faso, Huli, Huli-Foi, Kutubu, Baina) at HF and MSK are shown. The sequential rollout and number of operational MSK sites over time are also indicated.

### 3.2. Number of Cases Reported

During the study period 15,726 individuals were tested with either RDT, microscopy or (usually) both. Of those tested in health facilities, (42%) had a positive result reported for malaria. In those individuals who tested positive, (67.1%) had a *P. falciparum* infection (alone or mixed with other species) and 32.9% were infected with non-*P. falciparum* species. 

### 3.3. Test Positivity Rate

**Figure 3:**
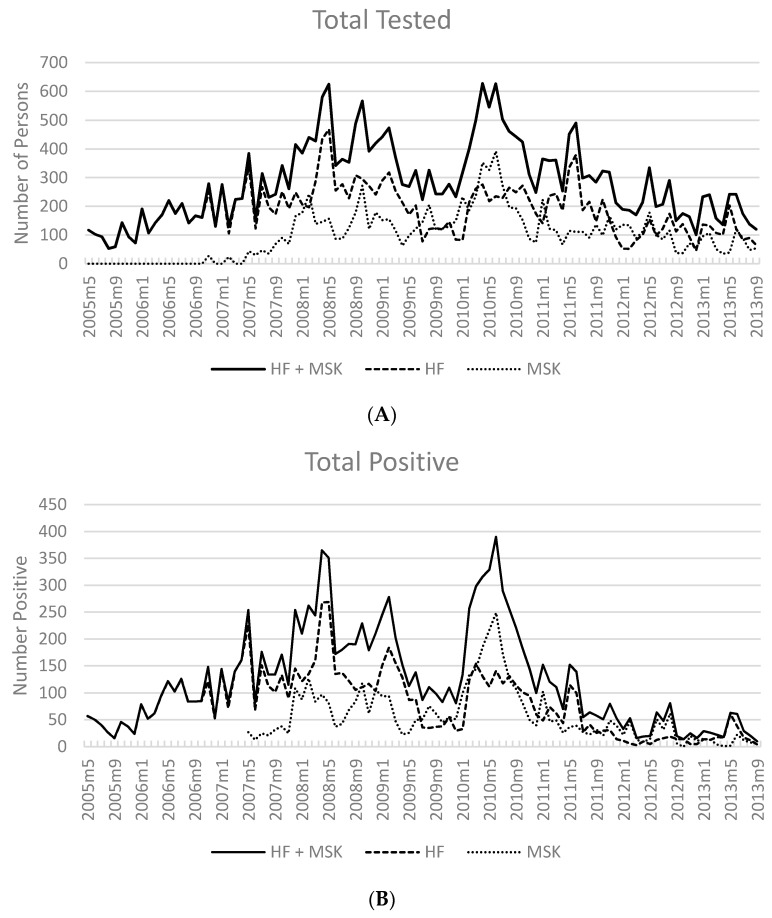
Number of persons tested with RDT and/or microscopy (**A**) at HF, MSK and HF + MSK; (**B**) number of persons with a positive test (October 2007–September 2013).

Figure 3A shows the monthly number of tests performed overall and by HF or MSK and the Figure 3B the reported number positive, for any malaria species by any test. Test positivity (any test) decreased from 57.7% to 53.4% overall from 2007 to 2010, (Table 1 and Table 2), a decline of 4.3%. From 2010 to 2013 test positivity (any test) decreased from 27.7% to 16.7%.

At HF, test positivity (any test) decreased from 58.9% to 48.6% overall from 2007 to 2010 (Table 1 and Table 2), a decline of 10.3%. In 2011, test positivity decreased to 24.2% with a further decline in 2012 to 9.9%. In 2013 test positivity increased to 18.6%. 

The proportion of individuals with a positive test at MSK sites from 2007 to 2008 (52% and 52.4%) declined to 43.1% in 2009. Test positivity decreased appreciably over the next three years, from 33.9% in 2011, to 25.7% in 2012, and to 13.5% in September 2013. 

### 3.4. Average Cases per Month by Facility Type

Of the 28,819 individual case records from both HF and MSK sites, 87.5% had an RDT result, 90.1% had a microscopy result, and 98.9% had either an RDT or microscopy result. The mean number of individuals tested (RDT, microscopy, RDT or microscopy), numbers of Pf or Pf mixed and non-Pf infections, were roughly double for all indicators at HFs (Table 3) in comparison to MSKs per month (Table 4). All blood smears were collected from HF and MSK and analysed at the OSL PHU lab as part of routine surveillance and quality assurance practices. 

The overall average number of persons tested and confirmed cases per month by malaria species, as assessed by the different methods in each type of site, is shown in Table 3 and Table 4. It can be seen that the numbers tested and positive by the two diagnostic methods were relatively similar, since the great majority of persons were tested by both methods. The concordance between diagnostic test results is being analysed separately. 

The overall number of positive results reported for malaria from May 2005 to September 2013 at HF and MSK sites by month is shown in Figure 3A. The patterns were different at HF and MSK sites, with more cases presenting to HF in the early years and more at MSK in the latter part of the study period. A clear annual cycle of malaria transmission was not evident, although intermittent increases of cases could be seen in early 2008 and mid-2010. 

### 3.5. Species Distribution

**Figure 4:**
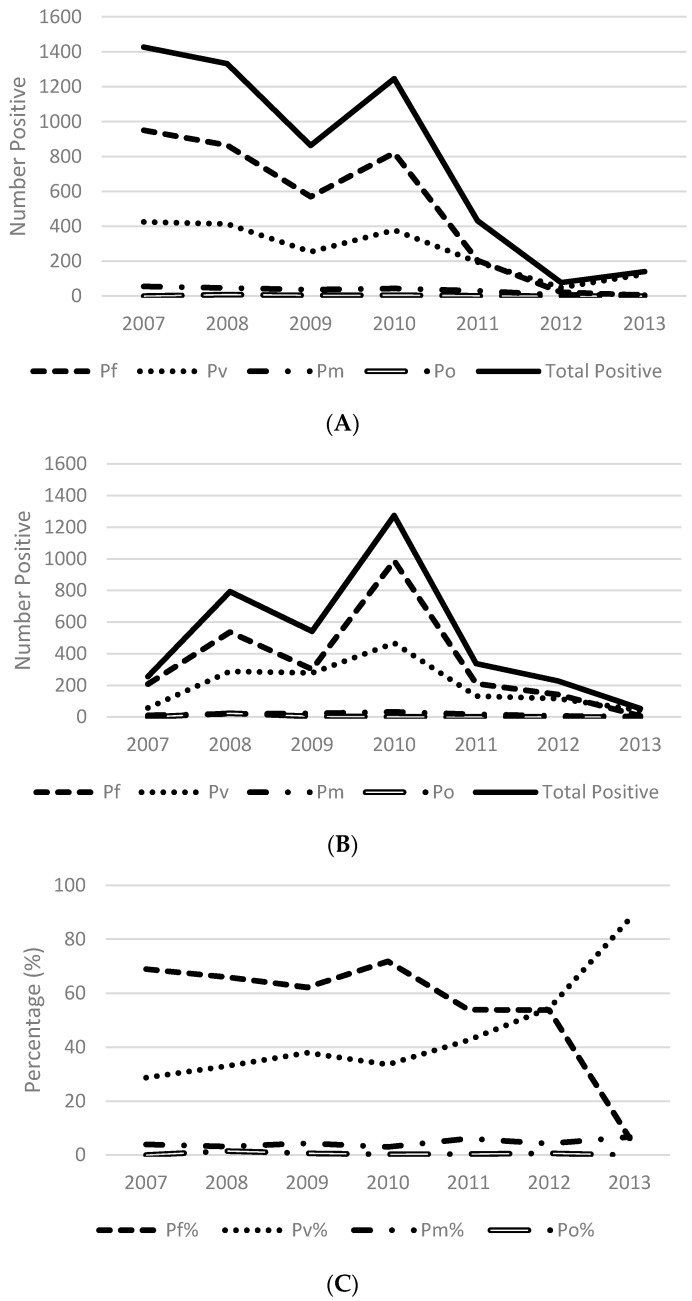
Malaria species distribution by year Kutubu, Southern Highlands Province, PNG. (**A**) Species distribution at HF; (**B**) species distribution at MSK; (**C**) proportion of infections attributed to infecting species at HF and MSK combined.

The distribution of *Plasmodium* infections by species per year was determined by microscopy (HF and MSK) and is presented in Figure 4. Species distribution was defined as results for overall positivity (any species), Pf (Pf, Pf gametocytes, Pf mixed), Pv (Pv, Pv mixed), Pm and Po.

The overall distribution of infections by species in individuals testing positive for malaria at HF sites by year is shown in Figure 4A. The predominant infecting species identified at HF was *P. falciparum* from 2007 to 2011. During this period, the percentage of infections that were Pf or Pf mixed declined from 73.9% to 51.1% (number of cases range 5 to 950). In 2012 and 2013 the percentage of Pf and Pf mixed infections further decreased to 39.7% and 19.6% respectively. Conversely, the contribution of non-Pf species infection to the overall burden of malaria gradually increased over the study period from 404 individuals (26.1%) in 2007 to 156 individuals (80.4%) in 2013 based on a much smaller total number of infections.

The overall distribution of infections by species in individuals testing positive for malaria at MSK sites by year are shown in Figure 4B. In the 4283 individuals who tested positive, 2796 had a Pf or Pf mixed result (65.3%), while 1487 had a P other result (34.7%). 

In the first four years of the MSK program the number of MSK sites increased from six to 13 and the access to EDAT improved. As a result, the observed number of reported infections increased in conjunction with enhanced case detection. Throughout this period a rise in the number of malaria infections was reported at MSK sites until mid-2010, when an appreciable decline in infections (all species) was observed. During the initial rollout phase from 2007 to 2010 malaria infections attributed to *P. falciparum* (range 209 to 988) and *P. vivax* (range 58 to 468) were most common, with *P. falciparum* the most dominant species identified (Figure 4B). Infections attributed to Pm (range 11–33 cases) and Po (range 0–24) were the least common. While both *P. falciparum* and *P. vivax* infections decreased from mid-2010 until 2013, a shift in species dominance was also observed at MSK sites from *P. falciparum* to *P. vivax*. 

Figure 4C depicts the proportion of infections attributed to *P. falciparum*, *P. vivax*, *P. malariae* and *P. ovale* from data collected at both HF and MSK sites over time. The dramatic shift in the proportion of infections attributed to *P. vivax* (88%) in 2013 is illustrated.

### 3.6. Overall Malaria Incidence in Kutubu (May 2005–September 2013)

**Figure 5:**
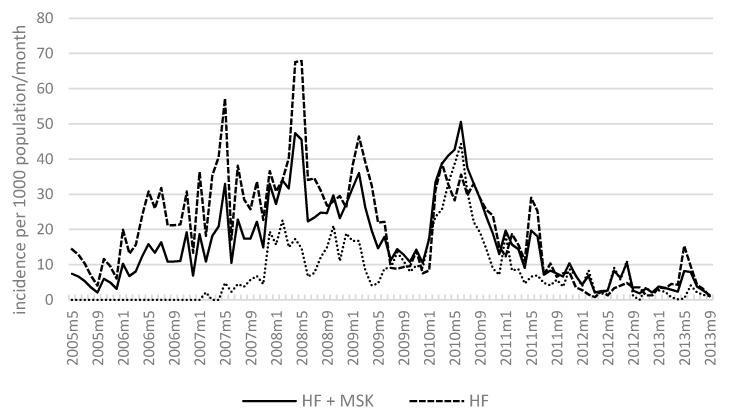
Malaria incidence (reported cases per 1000 population per month) at HF, MSK and both sites combined (May 2005–September 2013).

A summary of monthly incidence data (confirmed cases per 1000 population) from HF and MSK catchment areas is provided in Figure 5. HF data cover the period from May 2005 to September 2013 while MSK data cover February 2007 to September 2013. The combined data showed an increasing trend in incidence from 2005 to 2008 that peaked in April 2008. Incidence then decreased until December 2009, with the exception of an upsurge from December 2008 to February 2009. 

At the beginning of 2010, a generalised outbreak occurred, marked by a six-month period of high incidence that peaked from December 2009 (10.5 per 1000 per month) to the highest recorded peak in April 2010 (50.57 per 1000 per month). Over the ensuing six months, overall incidence decreased to 12.96 per 1000 per month, with intermittent minor peaks occurring in January, May and June 2011. Overall incidence levels continued to decline (range 1.29 to 10.5 per 1000 per month) with intermittent peaks until September 2013, when the lowest incidence was recorded (1.29 per 1000 per month).

### 3.7. Malaria Incidence: Health Facilities and MSKs

Summaries of monthly incidence data collected at HFs and MSKs are also provided in Figure 5. Peaks in incidence typically occurred between December to June, which corresponds to the months preceding and during a wetter season (March to September). In comparison to HF, incidence at MSK sites (range 1 to 44 per 1000 per month) was generally lower than HF (range 2 to 68) overall. 

At HF, incidence increased from 2005 to 2010, followed by a decline from mid-2010 to 2013. Within this timeframe there was an increasing trend of incidence, with sporadic peaks from 2005 to 2008. From January 2010, incidence reached a max peak in June 2010 (50 per 1000 per month) and steadily declined to September 2013, with the exception of two peaks observed in May and June 2011 and May 2013. At the final point in our analysis in September 2013, incidence was three per 1000 per month, the lowest reported during the study.

Similar to HF, incidence at MSKs increased overall with irregular peaks from 2007 to 2010 followed by a reduction from mid-2010 to 2013. The maximum peaks at MSK were recorded between January and April 2010 (range 18 to 44 per 1000 per month). Following April 2010 incidence declined over the next three years up to September 2013 (range 1 to 30 per 1000 per month), with notable peaks in January 2011 (18 per 1000 per month) and August 2012 (11 per 1000 per month). 

## 4. Discussion

This report describes the epidemiology of malaria in Kutubu and examines the diagnostic coverage of the MSK program over a 6.5-year period. Prior to the inception of the MSK, contemporary information on malaria in the Highlands of PNG was provided by the surveys conducted by the PNG IMR in 2003–2004 and in 2008–2009 [6,10,11,23,24,25,26,27,28,29]. The data presented here provide an overview of the MSK program and a monthly chronological picture of malaria epidemiology following the PNG IMR surveys in Kutubu up to September 2013. 

The results of this study indicate an overall large reduction in malaria during that time, and a shift toward predominance of *P. vivax*. Where malaria transmission is dramatically reduced, it is expected that there would be a shift at least in the short term towards *P. vivax*, since relapse infections can occur from dormant liver stages. In the longer term, *P. vivax* incidence should also decline if transmission is reduced. A shift in age-specific incidence towards older age groups would also be expected and is being investigated separately. 

A substantial body of literature describes the behavioural patterns of care-seeking treatment for malaria, although primarily in the African context [30,31,32,33,34]. The selection of provider for treatment is multifaceted and influenced by links between illness and individual provider characteristics [34,35]. Factors that influence treatment-seeking behaviour include past experience with malaria and perceived treatment outcome [36], gender [37], family size and occupation [38,39], age [32], education and socioeconomic status [33]. Treatment providers encompass an extensive range of formal and informal entities including public health facilities, private health facilities, faith-based organisations, village health workers [40], medicine vendors [41], traditional healers and sharing within family units and communities. 

Of the few studies concerning treatment-seeking behaviour in PNG, investigators have identified several factors that support the existing literature. Distance from a health facility [42,43,44], sex, age, cost, illness severity, perceived effectiveness of treatment and past experience with malaria contribute to what treatment is sought [42,43,45]. In a study examining two culturally and demographically distinct regions, Davy et al. [41] found that individuals seeking treatment for malaria in Madang and Maprik preferred public health centres over private retailers (pharmacies, kiosks, shops) based on accessibility and perceived quality of service. While cost did not factor into preference, Davy et al. suggest that provider choice was predicated on the belief that quality medicines were more likely available at public facilities [42]. 

In the current study we also observed a larger number of individuals seeking treatment at government-operated and faith-based HF (*n* = 15726) in comparison to MSK (*n* = 9687) over time. However, unlike Davy et al., whose study did not include village-based health workers, our data suggest that a significant proportion of people access care outside of these public facilities for treatment of malaria in Kutubu. 

Overall diagnostic metrics for confirmed malaria by RDT, microscopy or both indicate that the mean number of patients tested per month at MSK was half that tested at HF in 620 observation months (Table 2). The proportion of individuals tested overall (HF + MSK) increased from 18% to 36% at the MSK in the first year (2007–2008) and ranged from 36% to 49% throughout the study. However, during periods of peak incidence in 2008 and 2010 there was a concurrent and marked rise in the number of individuals tested at the MSK, perhaps reflecting increasing trust in the availability and perceived quality of service provided by MSK. In 2010 in particular, during a generalized outbreak, the numbers of individuals seeking treatment at the MSK were comparable to HF (Table 1 and Table 2). 

When using the number of patients tested (RDT, microscopy or both) as a metric of service “use” by ethnic cluster (Figure 2) treatment-seeking at the MSK was impacted by distance from a HF, availability of an operating HF, and or during periods of outbreak. 

The Kutubu, Huli and Huli-Foi clusters provide an interesting stratification of treatment-seeking behaviour based on HF operation in each region. At least one highly functional HF operates in each of these clusters and overall the number of patients tested at these facilities was higher than the MSK (Figure 2B–D). In areas where a highly functional HF provides service to the community, as in the Kutubu area, MSK use is low (Figure 2D). In the Huli cluster, three HFs provide services and the patient use of public facilities is also higher. However, the Huli people are highly mobile, which increases the risk of malaria introduction; civil unrest often leads to public service disruption. MSK use in this cluster is higher than in Kutubu despite close proximity to HF. Whether these factors underpin MSK use in this area needs to be determined via qualitative analysis. 

Additionally, the Yalanda MSK in the Huli cluster is located in an isolated mountain village only accessible on foot where no public service provision is available. The MSK has operated in this village without interruption since 2007. Similar findings are reported in the village of Baina, where the MSK is only provider for malaria treatment. 

In areas where public service provision is compromised, the MSK can serve as a viable and quality service provider option. In the Faso area, diagnostic services at the local HF were halted from 2006 to 2011. During this time, four MSK sites were established and 2068 individuals were tested (Figure 2A). Following the reopening of the HF in 2011, patients continued to access the MSK service. Similarly, in the Huli-Foi cluster, the Kaipu HF was closed from August 2008 to November 2011. The Kaipu MSK that opened in July 2008 tested 396 individuals during this period and continued to operate following the reintroduction of services. These findings suggest the local belief in the quality of care by the MSK, as suggested by Davy et al. [42]. Qualitative analysis of social determinants and treatment-seeking behaviour regarding the use of the MSK service is required. 

In a recent study examining the economic determinants of provider choice in a rural PNG setting, Tsukahara et al. found that distance, drug availability and severity of illness increased the probability of using a non-professional, village-based health worker [45]. Similar factors may have contributed to the increase and sustained use of the MSK. 

Key aspects of the MSK implementation strategy potentially mitigated the negative impact of distance, cost, quality-assured testing and drug availability that are known barriers to seeking treatment. MSKs were developed in malaria endemic communities thereby reducing travel distance, associated costs of transit and availability of EDAT. OSL field officers routinely visited (fortnightly) MSK villages to ensure supply stocks of RDTs, blood slides and antimalarials. Given that the MSK is an elected community member and business person, this information would be disseminated. Ongoing community engagement, health education and advocacy by OSL field teams would strengthen familiarity and the perception of quality around MSK service provision. While our study is descriptive, further qualitative studies using mixed methods analysis including household surveys of provider choice and perception of the MSK are needed to confirm these assumptions. 

From 2007 to 2010 we observed intermittent peaks in the incidence of malaria. The incidence variation is probably due to several factors:

(1) the sequential increase in the number of MSK sites and thus increased case detection
(2) climate variation
(3) population mobility and economic development

The movement of populations along highways that traverse endemic areas allows people and malaria parasites to be introduced into regions with differing malaria ecology [46]. A historical precedent exists around movement, malaria transmission and economics in the SHP that is facilitated by travel along the highlands highway to the endemic coastal provinces of Madang and Morobe [47,48]. Mueller et al. (2002) found that travel to lowland coastal communities to market highland vegetables was a significant risk factor for infection with *P. falciparum* in areas that are otherwise only climactically suited for endemic *P. vivax* transmission [47]. In a local context, Maraga et al. (2011) reported that population mobility, an integral lifestyle component of Southern Highland populations, likely contributed to the high prevalence (17%–33%) of malaria in Kutubu. In their study, over 90% of participants reported regularly sleeping in the bush and travelling to lower elevations for hunting and agricultural purposes in endemic areas [10]. Anecdotally, the Huli ethnic group in Kutubu are known to keep this practice, as established Huli gardens and settlements are found along the eastern shoreline of Lake Kutubu, traditionally home to the Foi ethnic group (Figure 1). 

An example of this theme of economics and population mobility is illustrated in our study. In 2010, an influx of migrants to OSL project areas was spurred by economic development and the large scale construction of the PNG liquefied natural gas (LNG) pipeline. Motivated by a burgeoning work force and surrounding communities now infused with disposable income, mobile populations sought economic benefits from services provided to these entities. We observed a marked increase in the number of patients tested for fever and diagnosed with confirmed malaria in the Huli-Foi area, the primary construction zone at the time (Table 1 and Table 2, Figure 1 and Figure 3A). In response to the rise in confirmed malaria, a fourth MSK in Tagresere was opened in 2010 to accommodate the volume of patients seeking treatment for fever. In total, four MSK sites tested (*n* = 556) and treated similar numbers of malaria, Pf and Pf mixed (*n* = 276) to those tested (*n* = 626) and treated, Pf and Pf mixed (*n* = 320) at HF; an increase of 61% tested and 45% treated by the MSK from the previous year (2009). These results suggest that village based care provision could serve as an adjunct to HF management of outbreaks, particularly in remote areas. 

## 5. Conclusions

This study is the most recent longitudinal overview of malaria in the Southern Highlands since 2003. It outlines patient access to a community-based model of care. The analysis shows changes in health facility versus MSK use, a strongly decreasing trend in the incidence of confirmed malaria from 2010 to 2013, and a shift from predominantly *P. falciparum* to *P. vivax* infection.

The increased early case detection and treatment provided by the MSK may have contributed to the drop in malaria transmission and the shift towards *P. vivax*, but this cannot be confirmed directly based on this data from cases presenting to HFs and MSKs. This question is being examined independently using separate data from repeated village surveys over the same time period. Comparisons of microscopy and RDT, individual village prevalence surveys and age-specific incidence are being examined to further understand malaria in Kutubu. Additionally, it is recommended that qualitative research be undertaken to understand the social and economic factors that can lead to a more stable model for expansion as maintaining the salary for the MSK will be a challenge when malaria decreases. 

## Figures and Tables

**Table 1:** Total number of reporting sites, individuals tested and malaria species distribution at HFs by year (2007–2013).

Year	# HF	Reporting Months *	Total Tested	Total Pos	(%)	Pf or Pf Mix	(%)	P Other	(%)
2007	5	48/60	2623	1546	58.9	1142	73.9	404	26.1
2008	6	69/72	3470	1787	51.5	1266	70.8	521	29.2
2009	6	64/72	2122	1025	48.3	721	70.3	304	29.7
2010	7	67/84	2700	1313	48.6	897	68.3	416	31.7
2011	9	90/108	2541	614	24.2	314	51.1	300	48.9
2012	8	79/96	1227	121	9.9	48	39.7	73	60.3
2013 **	8	70/96	1043	194	18.6	38	19.6	156	80.4
Total			15726	6600	42.0	4426	67.1	2174	32.9

* Reporting defined as the total month records from sites in column 2 contributed in one year, i.e., each site can contribute up to 12 months per year; ** Data collected until September 2013.

**Table 2:** Total number of reporting sites, individuals tested and malaria species distribution at MSKs (2007–2013).

Year	# MSK	Reporting Months *	Total Tested	Total Pos	(%)	Pf or Pf Mix	(%)	P Other	(%)
2007	6	20/72	576	302	52.4	244	80.8	58	19.2
2008	7	68/84	1914	996	52.0	673	67.6	323	32.4
2009	11	80/132	1493	644	43.1	339	52.6	305	47.4
2010	13	123/156	2549	1488	58.4	1014	68.1	474	31.9
2011	14	129/168	1429	485	33.9	314	64.7	171	35.3
2012	13	116/156	1103	284	25.7	182	64.1	102	35.9
2013 **	12	83/144	623	84	13.5	30	35.7	54	64.3
Total			9687	4283	44.2	2796	65.3	1487	34.7

* Reporting defined as the total month records from sites in column 2 contributed in one year, i.e., each site can contribute up to 12 months per year; ** Data collected until September 2013.

**Table 3:** Mean reported confirmed malaria cases by month at HF (2005–2013).

Indicator	Mean	sd	N *
RDT num. tested	28.59	31.27	559
RDT num. pos Pf or Pfmix	8.66	14.13	559
RDT num. pos P other only	2.58	4.15	559
RDT num. pos any	11.24	17.24	559
MIC num. tested	30.67	32.47	559
MIC num. pos Pf or Pfmix	7.85	12.94	559
MIC num. pos P other only	3.79	5.32	559
MIC num. pos any	11.65	17.04	559
RDT or MIC num. tested	31.35	32.00	559
RDT or MIC num. pos Pf or Pfmix	9.67	14.97	559
RDT or MIC num. pos P other only	4.58	6.28	559
RDT or MIC num. pos any	14.23	19.91	559

* N = number of observation months.

**Table 4:** Mean reported confirmed malaria cases by month at MSK sites (2005–2013).

Indicator	Mean	sd	N *
RDT num. tested	14.89	15.93	620
RDT num. pos.Pf or Pfmix	4.35	7.43	620
RDT num. pos P other only	1.51	2.97	620
RDT num. pos any	5.86	9.19	620
MIC num. tested	15.88	16.19	620
MIC num. pos Pf or Pfmix	3.89	6.75	620
MIC num. pos P other only	2.01	3.15	620
MIC num. pos any	5.90	9.04	620
RDT or MIC num. tested	15.38	14.20	620
RDT or MIC num. pos Pf or Pfmix	4.73	7.58	620
RDT or MIC num. pos P other only	2.47	3.78	620
RDT or MIC num. pos any	7.20	10.37	620

* N = number of observation months.

## Supplementary Materials

The following are available online at www.mdpi.com/2414-6366/2/1/2/s1.
