# Supplementary material for: The Epidemiology of Malaria in Kutubu, Southern Highlands Province, Papua New Guinea, before and during a Private Sector Initiative for Malaria Control
Source: Trop Med Infect Dis. 2017 Feb 10;2(1):2. doi: 10.3390/tropicalmed2010002 (PMC6082053; doi:10.3390/tropicalmed2010002)
Supplement: Supplementary file 1 [file tropicalmed-02-00002-s001.pdf]

Supplementary Materials: The Epidemiology of Malaria in Kutubu, Southern Highlands Province, Papua New Guinea, before and during a Private Sector Initiative for Malaria Control

Marshall Feterl, Patricia Graves, Liesel Seehofer, Jeffery Warner, Peter Wood, Kevin Miles and Ross Hutton

Methods

Original RDT and microscopy records were categorized as follows before collapsing for analysis:

Original RDT results were recorded as follows:

Pf	any *P. falciparum* positive infection
Pf mixed	any *P. falciparum* positive infection mixed with any other species
P other	any *P. vivax*, *P. malariae* or *P. ovale* infection or a mixture of these non-*P. falciparum* infections
Negative	any negative result
Invalid	any invalid RDT
nil	no result recorded

Original microscopy results were recorded as follows:

Pf	any *P. falciparum* positive infection
Pf Gam	any *P. falciparum* positive infection with gametocytes
Pv	any *P. vivax* infection
Pm	any *P. malariae* infection
Po	any *P. ovale* infection
Pf + Pv	*P. falciparum* infection with *P. vivax* infection
Pf + Pm	*P. falciparum* infection with *P. malariae* infection
Pf + Po	*P. falciparum* infection with *P. ovale* infection
Pv + Pm	*P. vivax* infection with *P. malariae* infection
Negative	no infection
unreadable	unreadable or broken slide
nil	no record or missing result
